# Black discoloration of the knee articular cartilage in a patient with pigmented villonodular synovitis: A case report

**DOI:** 10.1002/ccr3.7894

**Published:** 2023-09-11

**Authors:** Mohammad Ayati Firoozabadi, S. M. Javad Mortazavi, Hesam Toofan, Marzieh Khalili, Mohammad Javad Shariyate

**Affiliations:** ^1^ Tehran University of Medical Sciences(TUMS), Hip and Knee arthroplasty, Joint Reconstruction Research Center, Imam Khomeini Hospital Tehran University of Medical Sciences Tehran Iran; ^2^ Hip and Knee arthroplasty Joint Reconstruction Research Center, Imam Khomeini Hospital Tehran University of Medical Sciences Tehran Iran; ^3^ Hamadan University of Medical Sciences Hamedan university of Medical Science Hamedan Iran; ^4^ Department of Orthopaedic Surgery, Center for Advanced Orthopaedic Studies, Beth Israel Deaconess Medical Center Harvard Medical School Boston Massachusetts USA

**Keywords:** black cartilage, black menisci, case report, PVNS, total knee arthroplasty

## Abstract

In this case report, total knee arthroplasty was performed in a patient with pigmented villonodular synovitis. During surgery, severe black discoloration of the articular cartilage and menisci was observed in the patient. According to literatures, this is the first case report of severe articular cartilage pigmentation in a patient with pigmented villonodular synovitis.

## 1 INTRODUCTION

Mild discoloration of the articular cartilage is widely accepted to occur with age. However, pathological pigmentation of the articular cartilage and meniscus can occur in certain conditions in patients, such as alkaptonuria (ochronosis),^1^, ^2^ chronic phenol poisoning,^3^ artifacts,^3^ burns,^3^ hemosiderosis,^4^ Parkinson's disease medications,^5^ some antibiotics,^6^, ^7^ and hydroquinone.

In the present case report, total knee arthroplasty (TKA) was performed in a patient with pigmented villonodular synovitis (PVNS). During the surgery, severe black discoloration of the articular cartilage and menisci was observed. To our knowledge, this is the first report of black pigmentation of the articular cartilage and menisci in a patient with PVNS.

## 2 CASE REPORT

The patient was a 64‐year‐old woman with a 2‐year history of right knee pain and swelling without any traumatic injury. Physical examination revealed joint line tenderness and limited range of motion; however, despite conservative treatment, knee pain and swelling persisted. Clinical evaluation revealed acceptable hip‐knee‐ankle alignment. The patient had diabetes mellitus and received metformin.

**FIGURE 1:**
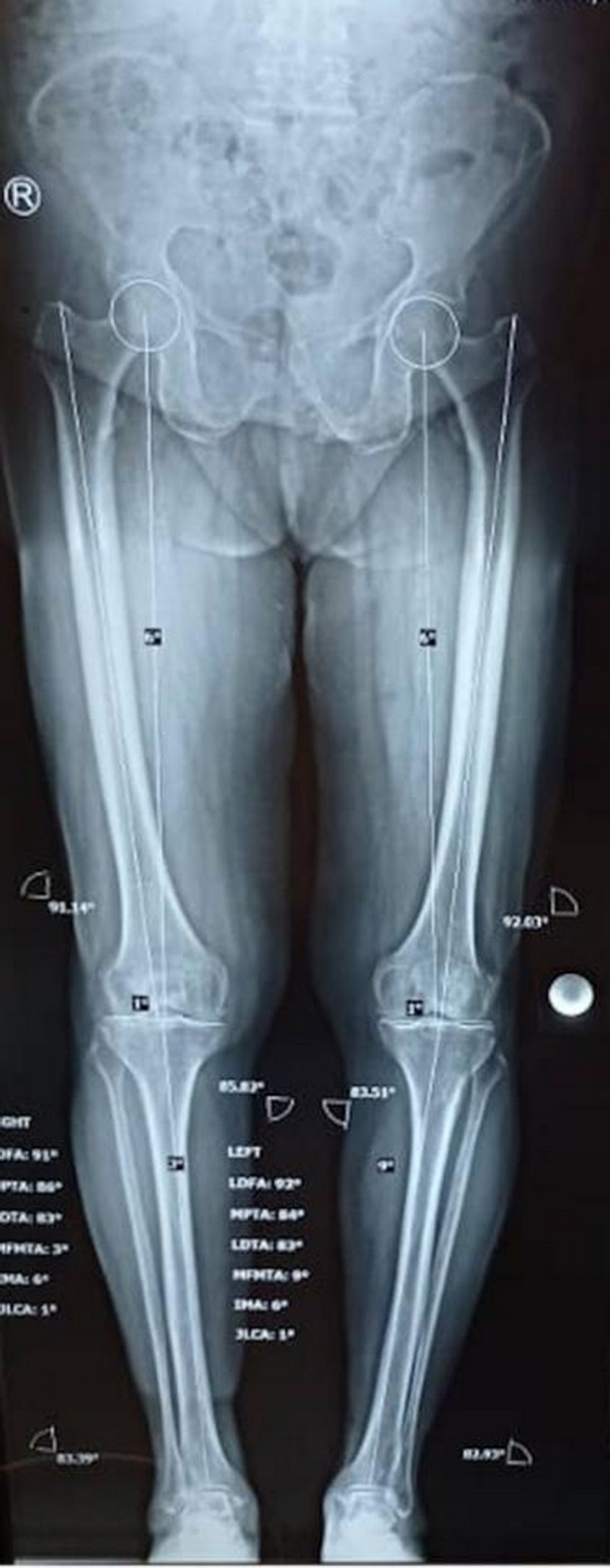
Hip‐knee‐ankle radiographic with joint space narrowing, subchondral sclerosis, and osteophyte formation.

Radiographic evaluation revealed joint space narrowing, subchondral sclerosis, and osteophyte formation (Figure 1). Furthermore, complete blood count values; erythrocyte sedimentation rates; C‐reactive protein, alanine transaminase, and aspartate transaminase levels; and activated partial thromboplastin and prothrombin times were all within the normal range. Physical examination revealed that a part of the patient's knee was swollen and had a rubbery consistency; therefore, knee magnetic resonance imaging (MRI) was performed to detect a possible lesion.

**FIGURE 2:**
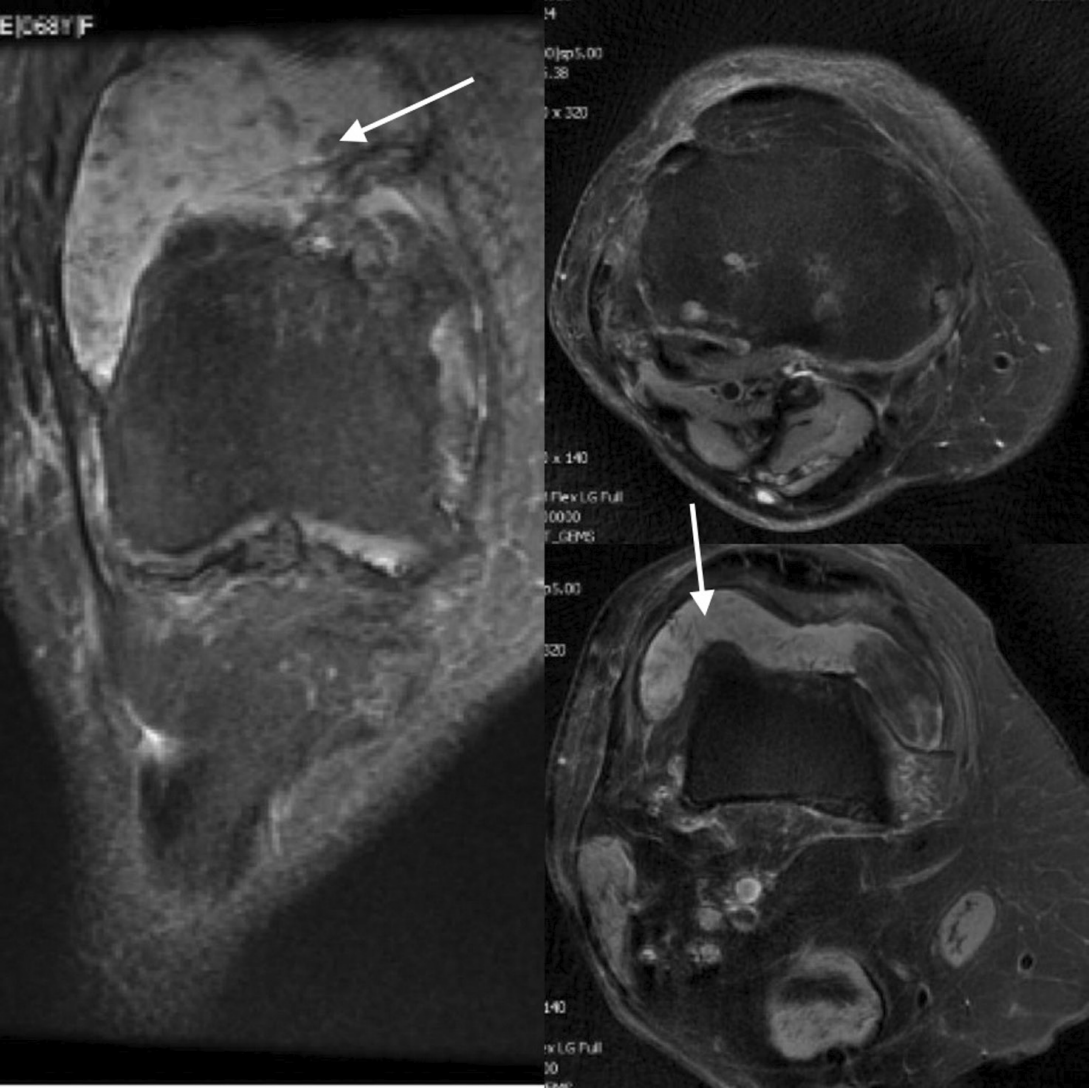
Knee joint effusion and popliteal cyst with internal debris which located posteriorly, suggesting the possibility of pigmented villonodular synovitis (PVNS).

MRI showed knee joint effusion with a popliteal cyst and internal debris located posteriorly, suggesting the possibility of PVNS (Figure 2).

Although there are different treatment options for PVNS, TKA with total synovectomy was selected owing to the patient's age and presence of cartilage destruction on radiography and MRI.

**FIGURE 3:**
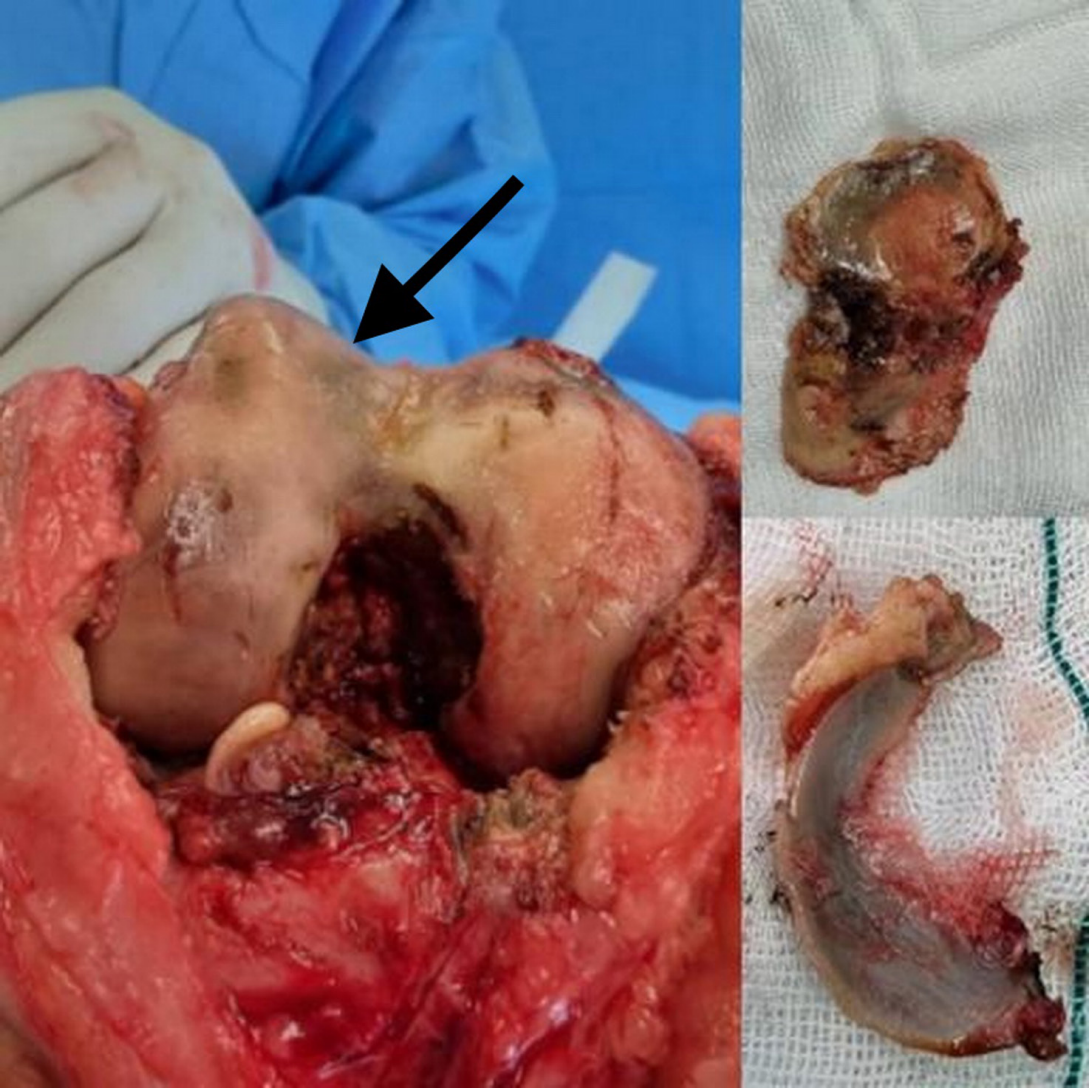
Severe black pigmentation of the synovial membrane and menisci and pigmentation of the articular surface.

Cemented right TKA was performed under spinal anesthesia by using a cemented posterior‐stabilized prosthesis (Stryker Triathlon® Total Knee System). The right knee articular cartilage was exposed using the medial parapatellar approach, which revealed severe black pigmentation of the synovial membrane and menisci, and pigmentation of the articular surface (Figure 3). The articular surface was eroded, and a small part of the subchondral bone of the medial femoral condyle was visible. After the femoral condyle was cut, pigmentation was found to be localized in the cartilage layer. Total synovectomy was performed, and the synovial and popliteal fossa mass biopsy specimens were analyzed.

**FIGURE 4:**
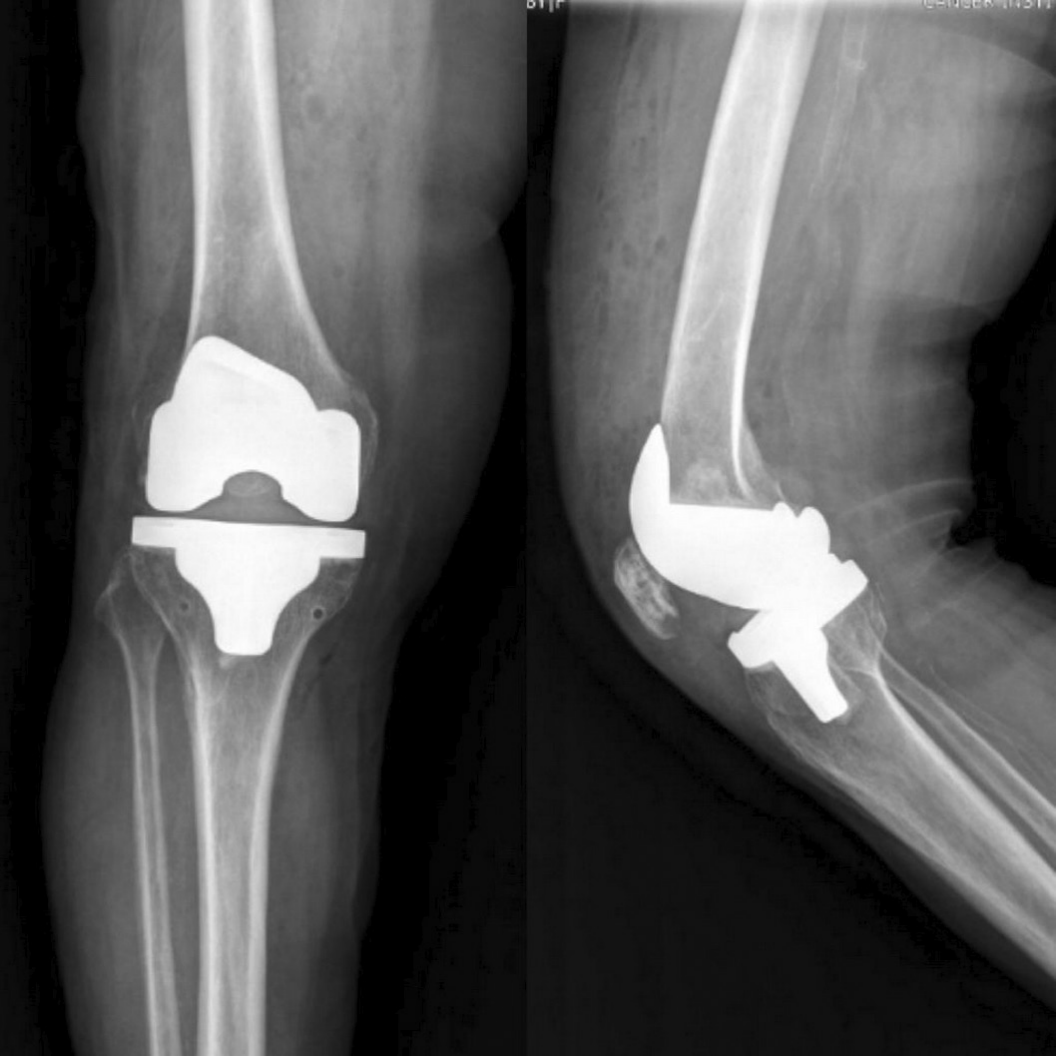
Postsurgical radiographic with acceptable alignment.

Postsurgical radiographic and clinical examination results were acceptable, and there were no intraoperative or perioperative complications (Figure 4).

**FIGURE 5:**
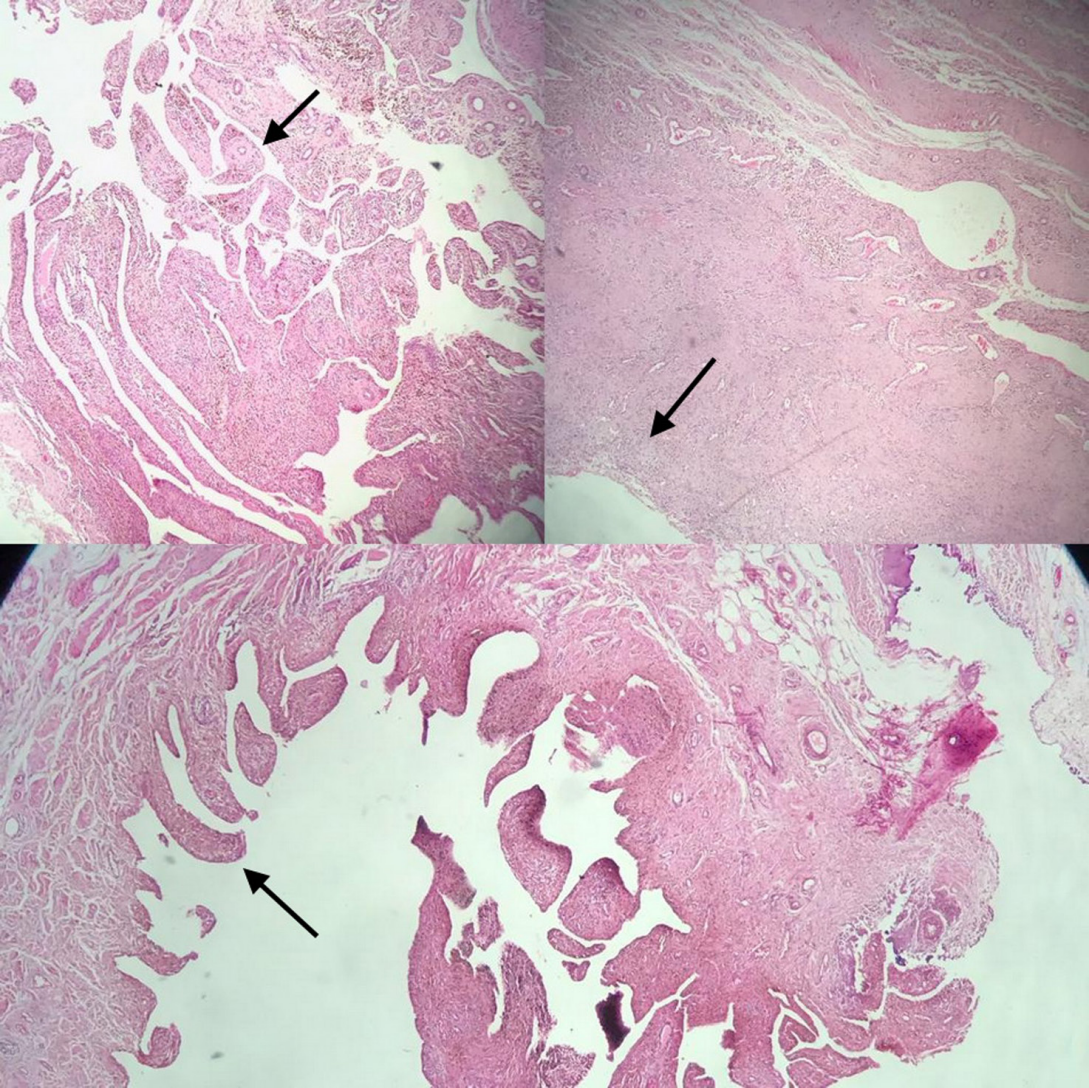
The lesion is composed of papillary, villous, nodular, and pseudoglandular or cleft like spaces with a synovial lining. Osteoclast‐like multinucleated giant cells and hemosiderin‐laden macrophages are seen. Histopathologic evaluation is compatible with pigmented villonodular synovitis (PVNS).

Diagnosis was confirmed by histopathologic evaluation, which revealed a tenosynovial giant cell tumor (PVNS; Figure 5).

According to the literatures, this is the first report of severe articular cartilage pigmentation in a patient with PVNS.

The patient was followed up for 14 months, during which time she was free of complications.

## 3 DISCUSSION

Pigmented villonodular synovitis is a proliferative disorder of the synovium with locally aggressive nature, and its etiology is unclear, although neoplasm, recurrent hemorrhage, and trauma have been reported as possible causes.^8^, ^9^ To our knowledge, there are no reports of severe articular surface, synovial, and meniscal pigmentation in patients with PVNS; however, certain conditions have been reported to cause severe articular cartilage and meniscal pigmentation abnormalities in patients, such as alkaptonuria (ochronosis) and use of antibiotics and drugs such as levodopa and methyldopa.^1^, ^2^, ^3^, ^4^, ^5^, ^6^, ^7^

In 2016, Matsumoto et al. reported black discoloration of the knee articular cartilage after spontaneous recurrent hemarthrosis in a patient and concluded that hemosiderin and lipofuscin accumulation due to recurrent hemarthrosis might increase black pigmentation of joint cartilage.^9^ They roll outed specific condition by histopathological examination.^9^

Patients with PVNS commonly present with hemarthrosis^10^; therefore, it is reasonable that recurrent hemarthrosis in patients with PVNS could cause articular surface and meniscal pigmentation owing to hemosiderin and lipofuscin accumulation and combination.

## 4 CONCLUSION

To our knowledge and according to the literatures, this is the first report of severe black pigmentation of the articular cartilage and menisci in a patient with PVNS. Based on report of black discoloration of the knee articular surface by Matsumoto et al,^9^ we hypothesize that recurrent hemarthrosis in patients with PVNS could result in severe articular and meniscal pigmentation owing to hemosiderin and lipofuscin accumulation.

## AUTHOR CONTRIBUTIONS

**Mohammad Ayati Firoozabadi:** Conceptualization; project administration; writing – review and editing. **S. M. Javad Mortazavi:** Supervision. **Hesam Toofan:** Data curation. **Marzieh khalili:** Data curation; visualization. **Mohammad Javad Shariyate:** Writing – original draft.

## FUNDING INFORMATION

This study had no financial support.

## CONFLICT OF INTEREST STATEMENT

The authors declare that they have no conflict of interests.

## CONSENT STATEMENT

Written informed consent was obtained from the patient to publish this report in accordance with the journal's patient consent policy.

## Data Availability

The data that support the findings of this study are available from the corresponding author upon reasonable request. More detailed data are available from the corresponding author upon reasonable request.
